# Etymologia: ***Cryptococcus gattii* [krip′′to-kok′әs ga-te-i]**

**DOI:** 10.3201/eid1602.ET1602

**Published:** 2010-02

This yeast genus takes its name from the Greek *kryptos*, hidden, and *kokkos*, berry. This pathogen has been recently recognized as a distinct species that causes infection (with cutaneous, pulmonary, and neurologic manifestations) in both humans and animals. The species was named for Italian mycologist Franco Gatti who, with Roger Eeckels, described an atypical strain of *C. neoformans* in the cerebrospinal fluid of a Congolese Bantu boy with cryptococcosis in 1970.

